# Supplementary material for: Abundance and Diversification of Repetitive Elements in Decapoda Genomes
Source: Genes (Basel). 2023 Aug 15;14(8):1627. doi: 10.3390/genes14081627 (PMC10454600; doi:10.3390/genes14081627)
Supplement: Supplementary file 1 [file genes-14-01627-s001.zip › genes-2521526-supplementary.pdf]

Genomes	contig N50	Number of scaffolds	Scaffold N50	Type of reads
Penaeus chinensis	470.2 kb	1060	36.9 Mb	L
Penaeus indicus	463.4 kb	11166	34.4 Mb	L+S
Penaeus japonicus	132.8 kb	18210	234.9kb	L+S
Penaeus monodon	45.2 kb	26875	44.9 Mb	L+S
Penaeus vannamei	86.9 kb	4682	605.6 kb	L+S
Caridina multidentata	819 bp	2750712	819 pb	S
Macrobrachium nipponense	267.3 kb	24	83 Mb	L
Panulirus oranatus	5.4 kb	403881	8.1 kb	S
Procambarus virginalis	12.2kb	169498	144.4 kb	L
Procambarus clarkii	217.7 kb	24238	17 Mb	L
Cherax destructor	80.9 kb	98662	87.2 kb	L+S
Cherax quadricarinatus	3.3 kb	508682	33.2 kb	L+S
Homarus americanus	133.3 kb	47245	759.6 kb	L+S
Paralithodes camtschaticus	5.8 kb	859811	7 kb	S
Paralithodes platypus	147.8 kb	6958	51.2 Mb	L
Birgus latro	5.3 kb	767134	6.3 kb	S
Chionoecetes opilio	149.6 kb	26514	208.1 kb	L+S
Eriocheir sinensis	3.2 Mb	4311	17.6 Mb	S+O
Portunus trituberculatus	4.1 Mb	523	21.8 Mb	L+S
Callinectes sapidus	9.3 kb	3967	18.8 Mb	L+S+O
Amphibalanus amphitrite	536.8 kb	/	/	L+S
Armadillidium vulgare	38.4 kb	43541	51.1 kb	L+S
Daphnia magna	1.5 Mb	308	12.5 Mb	L+S
Darwinula stevensoni	38.5 kb	62117	56.4 kb	M+S
Eurytemora affinis	67.7 kb	6171	252.3 kb	S
Hyalella azteca	112.9 kb	17395	213.8 kb	S

Table S1. Assembly metrics. Contig and scaffold N50, number of scaffold and type of reads produced. L: long reads; S: short reads; O: optical mapping; M: mate pair reads.

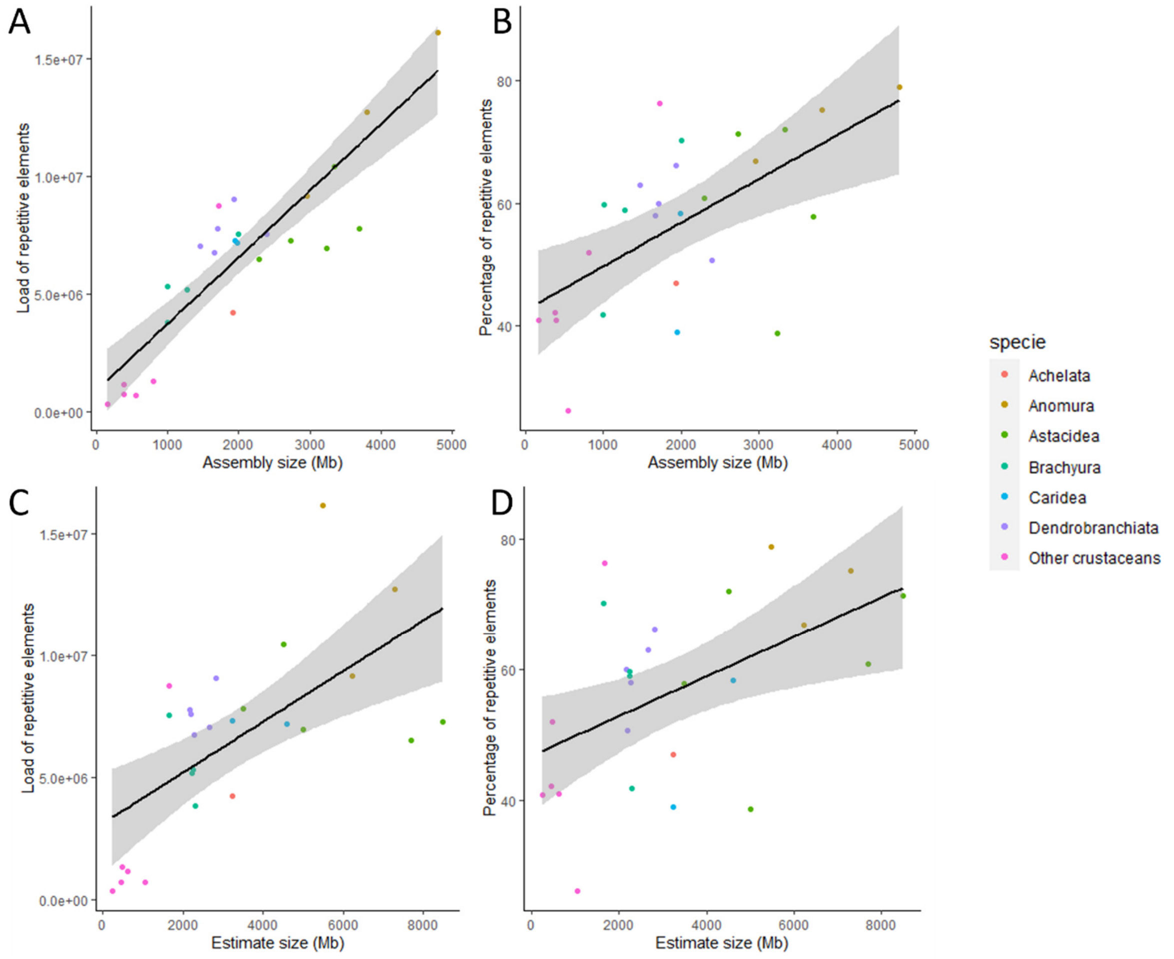

Figure S1. Correlation between genome size and REs. Correlation plot between assembly or estimate genome size and load or percentage of REs. Order and suborder are grouped by colours. A. Correlation between assembly size and the load of REs. Spearman rank correlation test: ρ=0.83, p-value=1.939E-6. B. Correlation between assembly size and the percentage of REs. Spearman rank correlation test: ρ=0.54, p-value=0.00502. C. Correlation between estimate genome size and the load of REs. Spearman rank correlation test: ρ=0.57, p-value=0.00208. D. Correlation between estimate genome size and the percentage of REs. Spearman rank correlation test: ρ=0.4, p-value=0.02745.

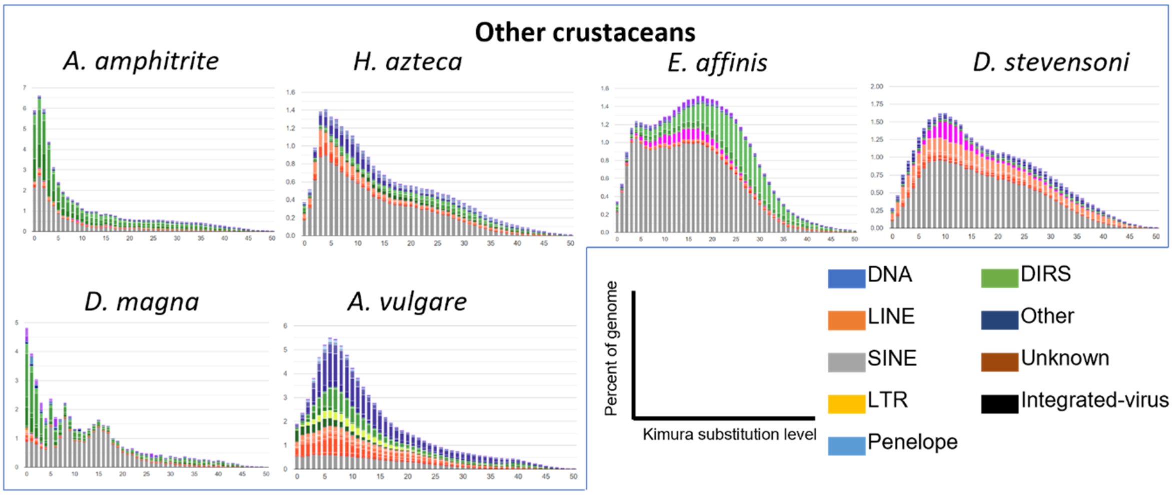

Figure S2. Sequence divergence distribution of TEs. TE accumulation history based on Kimura 2P distance. Sequence divergence on the x-axis. On the y-axis, the percentage of the genome represented by each TE type, the scale is different for each genome depending on the percentage occupied. TE type indicated by the colour chart.

Crustaceans non-Decapoda species studied present a large fraction of unknown elements in their sequence divergence distribution. Unknown elements can largely bias the analysis. Indeed, the identification of these unknown can change interpretation. *A. amphitrite* and *D. magna* present active TE with an expansion of LTR and unknown elements. In *D. magna*, we can also observe a peak of unknown elements at 15% of divergence. We can notice in *H. azteca* a peak at 5% of divergence of unknown elements. For *E. affinis* genome there is almost no distinction between the two peaks, around 4% of divergence and 10% to 30%. LTR can be the predominant elements of the oldest event, but an identification of unknown elements can change the interpretation. In *D. stevensoni* there is a peak between 5% to 10% of divergence of DNA transposons and unknown. *A. vulgare* present a high peak at a Kimura distance of 5% to 10% with an augmentation of the coverage of DNA transposons, LINE and LTR elements. At really low Kimura divergence, we can observe an increasing coverage of Penelope elements.
